# Infectivity of SARS-CoV-2 and Other Coronaviruses on Dry Surfaces: Potential for Indirect Transmission

**DOI:** 10.3390/ma13225211

**Published:** 2020-11-18

**Authors:** Max Bueckert, Rishi Gupta, Aditi Gupta, Mohit Garg, Asit Mazumder

**Affiliations:** 1 Department of Biochemistry & Microbiology, University of Victoria, 3800 Finnerty Road, Victoria, BC V8P 5C2, Canada; 2 Department of Civil Engineering, University of Victoria, 3800 Finnerty Road, Victoria, BC V8P 5C2, Canada; mgarg@uvic.ca; 3 Mearns Centre for Learning—McPherson Library, University of Victoria, 3800 Finnerty Road, Victoria, BC V8P 5C2, Canada; aditig@uvic.ca; 4 Department of Biology, University of Victoria, 3800 Finnerty Road, Victoria, BC V8P 5C2, Canada; mazumder@uvic.ca

**Keywords:** SARS-CoV-2, coronavirus, COVID-19, transmission, fomites, contaminated surfaces, persistence, stability, survival, disinfection

## Abstract

The unwavering spread of COVID-19 has taken the world by storm. Preventive measures like social distancing and mask usage have been taken all around the globe but still, as of September 2020, the number of cases continues to rise in many countries. Evidently, these measures are insufficient. Although decreases in population density and surges in the public’s usage of personal protective equipment can mitigate direct transmission of severe acute respiratory syndrome coronavirus 2 (SARS-CoV-2), indirect transmission of the virus is still probable. By summarizing the current state of knowledge on the stability of coronaviruses on dry materials, this review uncovers the high potential for SARS-CoV-2 transmission through contaminated surfaces (i.e., fomites) and prompts future research. Fully contextualized data on coronavirus persistence are presented. The methods and limitations to testing the stability of coronaviruses are explored, and the SARS-CoV-2 representativeness of different coronaviruses is analyzed. The factors which dictate the persistence of coronaviruses on surfaces (media, environmental conditions, and material-type) are investigated, and the review is concluded by encouraging material innovation to combat the current pandemic. To summarize, SARS-CoV-2 remains viable on the timescale of days on hard surfaces under ambient indoor conditions. Similarly, the virus is stable on human skin, signifying the necessity of hand hygiene amidst the current pandemic. There is an inverse relationship between SARS-CoV-2 surface persistence and temperature/humidity, and the virus is well suited to air-conditioned environments (room temperature, ~ 40% relative humidity). Sunlight may rapidly inactivate the virus, suggesting that indirect transmission predominantly occurs indoors. The development of antiviral materials and surface coatings would be an extremely effective method to mitigate the spread of COVID-19. To obtain applicable data on the persistence of coronaviruses and the efficiency of virucidal materials, future researchers should understand the common experimental limitations outlined in this review and plan their studies accordingly.

## 1. Introduction

The global spread of severe acute respiratory syndrome coronavirus 2 (SARS-CoV-2) has outpaced researchers’ attempts to develop an effective therapeutic drug or vaccine. For the time being, physical distancing has proved to be a somewhat effective non-pharmaceutical approach to minimizing viral transmission [1]. However, there is a mode of transmission which may elude such a measure: indirect contact through contaminated surfaces. 

A virus can be deposited onto a surface in multiple ways. Infected individuals may self-inoculate their appendage and touch a surface; public railings, handles, and other high-touch surfaces are common subjects to this mode of deposition. Alternatively, respiratory droplets may be expelled when infected people sneeze, cough, speak, etc. [2,3,4]. It has been observed that the large droplets are too heavy to remain airborne and will eventually fall, subsequently contaminating the surfaces below [5]. The virus spreads through ‘indirect contact’ when a new individual touches the contaminated surface. Thereafter, self-inoculation of the mucous membranes or serial surface transfers occur [2]. 

The occurrence of SARS-CoV-2 shedding into the environment has been made evident through RNA (i.e., coronaviruses’ genetic material)-based contamination assays [6,7,8]. Commonly, up to 50% of high-touch hospital surfaces tested positive for the presence of SARS-CoV-2 RNA [8]. Contamination of these surfaces demonstrates the occurrence of hand-to-surface inoculations. The several positive results in floor samples, as referred in Guo et al., are a testament to surface contamination via fallen respiratory droplets [6]. In a more recent study (preprint), positive results were obtained from 29/348 (8.3%) samples collected from high-touch surfaces in public locations and essential businesses (e.g., crosswalk buttons or liquor store door handles) throughout a town in Massachusetts. Moreover, weekly positivity rates were strongly correlated with COVID-19 cases seven days later, suggesting environmental surveillance may be key to understanding and predicting coronavirus transmission [9].

Importantly, evidence of RNA contamination does not corroborate the existence of infectious virus particles. As discussed in the work of Atkinson and Petersen, polymerase chain reaction (PCR), which is used to detect genetic material, does not discriminate between infectious viruses and non-infectious nucleic acids. It is not uncommon for the RNA of SARS-CoV, MERS-CoV, Ebola, influenza, or Zika viruses to persist on surfaces for days to weeks after infectivity is lost [10].

Because a virus cannot replicate outside of a host, the chance of indirect transmission can only decrease following surface inoculation. The rate at which this probability decreases is dependent on the virus’s environmental durability [2]. There may be a misconception that SARS-CoV-2, like many enveloped viruses, cannot remain viable once it has lost its liquid medium. For example, Agrawal and Bharwaj modelled the evaporation dynamics of respiratory droplets deposited on solid surfaces under the assumption that once the media disappears, so does the infectivity of SARS-CoV-2 [11]. It is generally true that the lipidic nature of their membrane makes enveloped viruses more susceptible to desiccation and environmental stress but there are exceptions to this [2], and overlooking the persistence of SARS-CoV-2 could have severe implications on public health and policy [12].

To shed light on the potential for SARS-CoV-2 indirect transmission through contaminated surfaces, the present review summarizes the surface stability, that is, the duration of infectivity of SARS-CoV-2 and all other coronaviruses on dry surfaces. The methods, limitations, and data-applicability of the reviewed studies are addressed, the effects of inoculating media, ambient conditions, and material-type on coronavirus surface persistence are discussed, and the promising future of antiviral materials is explored. 

The following search terms were used in combination with “coronavirus,” “SARS-CoV,” or “COVID-19”: surface survival, surface stability, or surface persistence.

**Figure 1:**
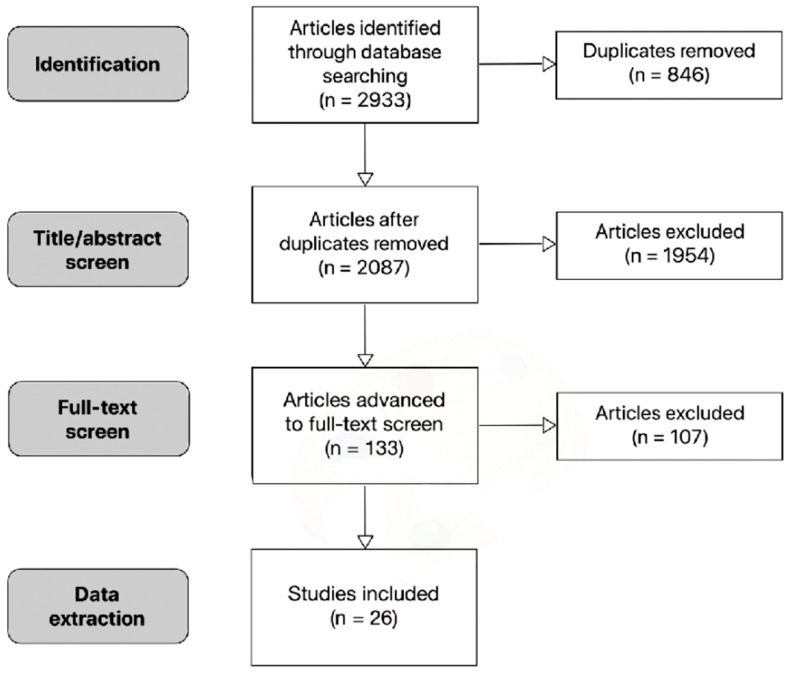
Flowchart of literature search.

The search was conducted on PubMed, Web of Science, ScienceDirect, and Google Scholar, which has been demonstrated to be a useful addition to other conventional databases due to its efficiency in the grey literature searches [13]. Screening the grey literature was considered an essential task because of the recent surge in SARS-CoV-2 research. However, typically only the first few hundred search results are relevant [13]. Therefore, the first 500 results on Google Scholar were screened. A total of 2087 studies were screened, and 26 relevant primary sources were found which met the search criteria, i.e., the studies contained data on infectious coronavirus titre reductions over certain amounts of time on specific surfaces in the absence of chemical disinfectants (see Figure 1).

## 2. Methods and Limitations of the Reviewed Studies

The rampant spread of SARS-CoV-2 has prompted plenty of research, but it has also out-paced the peer review process. As of September 2020, most of the studies on SARS-CoV-2 surface persistence are preprints [14,15,16,17,18], letters-to-editors [19,20,21,22,23], or test-reports [24,25,26]. Regardless, there are ubiquitous limitations to the applicability of the studies’ data that are apparent if the research methodology is understood. 

Although there was variation across experiments, the reviewed studies were generally conducted by first procuring a virus and determining its infectivity. The latter step was most commonly achieved through a tissue culture infective dose assay (TCID_50_), which measures the amount of infectious virus in a certain volume of liquid (i.e., the viral titre). Subsequently, microdroplets of the assayed virus-laden media were deposited onto sterile test substrates (e.g., plastic or stainless steel coupons) and left to dry. At select sampling points, the virus particles were recovered by substrate immersion, thereby resuspending the virus in an extraction media. Lastly, the viral titre of the extraction media was assayed and compared to that of the initial droplet to discern the titre reduction. For example, an initial SARS-CoV-2 titre of 10^4^ TCID_50_/mL may reduce to 10^2^ TCID_50_/mL following some time on a dry surface. This corresponds to a two-log-unit or a 99% titre reduction. 

There were inevitable and/or avoidable limitations to the foregoing methodology. Each experiment had a specific lower limit of detection (LOD) at which infectious virus could not be discerned, and distinct sampling points which influenced data. Consequently, the recorded durations of viability are inexact and presented as a range in this review. The lower range value corresponds to the final sampling point at which infectious virus could be recovered from the surface (i.e., the titre was at, or above, the LOD at this point). The upper range value corresponds to the first sampling point at which virus could not be detected. For example, SARS-CoV-2 persisted on stainless steel for 72–96 h [18]. This means that infectious virus could be detected at time (*t* = 72 h), but the viral titre was below the LOD at t = 96 h (the sequential sampling point). Studies often failed to report the LOD and/or choice of sampling points, so the applicability of their data is questionable. Although still influenced by the LOD and sampling points, perhaps a more pertinent measurement of stability is achieved through estimating viral decay rates (half-lives) with models (e.g., Bayesian linear regression). This method is becoming more prominent, but dated studies almost always failed to do this.

In addition to the inevitable sampling point and LOD bias, the aforementioned methodology was commonly flawed in two ways. Firstly, the composition of the droplets used to inoculate materials was rarely a clinically relevant matrix. Rather, culture media was used. Such experiments have faced criticism because real-world deposition media (e.g., saliva or mucous) contain interfering substances (e.g., antimicrobial proteins) which often impair pathogen stability [27,28]. As will be discussed, coronavirus stability was always reduced when substrates were inoculated with clinically relevant matrices [18,22,23]. Secondly, because viral recovery was achieved through material saturation, even trace levels of remaining virus were recovered. Lower sensitivity and, thus, shorter durations of viability, would have been observed had swabbing methods been employed (which are more representative of hand–surface interactions) [16]. There are no available data on the transmissibility of coronaviruses from inoculated surfaces to hands. However, after five seconds of contact, 1.5–37.8% of influenza A, parainfluenza 3, and rhinovirus virions (all respiratory viruses) transfer to hands [29,30]. 

## 3. Similarities between SARS-CoV-2 and Other Coronaviruses 

Before presenting data and discussing the factors that influence the persistence of SARS-CoV-2, it is important to address the representatives of other coronaviruses and whether data related to the latter can be extrapolated to the novel virus. This preceding comparison is critical to readers’ interpretation of data, and to future researchers who want to study the persistence of SARS-CoV-2 but do not have access to high-level containment labs. Only three studies were found which experimentally compared the stability of a SARS-CoV with another coronavirus: SARS-CoV-2 versus SARS-CoV-1 [22], SARS-CoV-2 versus HCoV-299E and FCoV [18], and SARS-CoV-1 versus HCoV-299E [31]. Unfortunately, their results contradict one another. 

The findings of van Doremalen et al. suggest that the stabilities of SARS-CoV-2 and SARS-CoV-1 are quite similar. The viruses had comparable half-lives on polypropylene, stainless steel, and copper (SARS-CoV-2: 6.81 h, 5.63 h, and 0.77 h, respectively; SARS-CoV-1: 7.55 h, 4.16 h, and 1.5 h, respectively). Their seemingly dissimilar stabilities on cardboard were results of extremely variable data. The researchers suggested that differing environmental stabilities were not to blame for the viruses’ distinct epidemiological characteristics [22]. Taken together, however, the results of Szpiro et al. and Rabenau et al. challenge this [18,31].

The stabilities of two surrogate coronaviruses (HCoV-299E and FCoV) were compared to that of SARS-CoV-2. The prolonged viability of the surrogates under most conditions was indicative of the novel virus’s reduced durability [18]. On the contrary, a separate study found that SARS-CoV-1 persisted on polystyrene for six to nine days, whereas HCoV-299E could never be recovered by day three [31]. 

To summarize these discrepancies, the stabilities of SARS-CoV-2 and SARS-CoV-1 are similar [22], SARS-CoV-2 is less persistent than HCoV-299E [18], but SARS-CoV-1 is considerably more persistent than HCoV-299E [31]. Clearly, these contradictory findings warrant further investigation. Notwithstanding their potentially dissimilar durations of infectivity, however, there are common properties of all coronaviruses. Copper, a well-documented antimicrobial [32], rapidly inactivated SARS-CoV-2, SARS-CoV-1, and HCoV-299E [22,33]. Low temperatures and/or low relative humidity (RH) levels (<50%) favoured the persistence of SARS-CoV-2, SARS-CoV-1, MERS-CoV, TGEV, MHV, HCoV-299E, and FCoV [15,18,23,34,35,36,37]. Thus, the relative stabilities (response to different conditions) of different coronaviruses may be more consistent than their absolute stabilities (duration of infectivity under a certain condition). Antiviral materials and the effects of ambient conditions on coronavirus persistence will be expanded on in the following sections.

## 4. Effect of Media on SARS-CoV-2 Persistence 

The contents of the media used to dry viruses on substrates significantly influences viral stability. Most studies inoculated surfaces with SARS-CoV-2 in culture media. However, real-world deposition media (e.g., mucus) contains high levels of interfering substances like specific antibodies, leukocytes, antimicrobial proteins and peptides, competing microbes, etc., all of which can be disadvantageous to coronavirus persistence [27,38]; SARS-CoV-2 dried in culture medium persisted for 48–72 h on stainless steel at RT, but for only 30–48 h when dried in artificial saliva/mucous mix [18]. Similar results were obtained by van Doremalen et al.; in an initial experiment which utilized culture medium, the half-life of SARS-CoV-2 on plastic was 6.81 h [22]. However, a later experiment showed that when SARS-CoV-2 was dried in nasal mucus or sputum on plastic under comparable ambient conditions, the half-life reduced by over 50% (3.1 h) [23]. These findings suggest that future virus persistence research should be conducted exclusively with clinically relevant matrices. 

Prior to surface inoculation, many researchers added proteins like BSA or FCS to the culture medium to mimic the high protein levels found in respiratory fluids [20,21,31]. Ironically, these protein additives provided more stability to dried SARS-CoV-2 and SARS-CoV-1, and thus the effects of protein enriched culture media were opposite to the effects of real-world-deposition media demonstrated by Szpiro et al. and van Doremalen et al. [18,22,23]. When dried in culture medium, the half-life of SARS-CoV-2 was >96 h on polypropylene and 2.5 h on aluminum. However, following the addition of BSA (10 g/L) to the culture medium, the effect of surface type was concealed; the half-life of SARS-CoV-2 was >96 h on aluminum and polypropylene alike [21]. In a separate study, the addition of 10% FCS to the culture medium slightly enhanced the stability of SARS-CoV-1 dried on polystyrene as well, albeit less so [31]. Interestingly, SARS-CoV-2 dried in a tripartite soil load containing BSA, mucin, and tryptone could persist for >21 days [16], which is the longest duration of SARS-CoV-2 infectivity recorded in this review. 

The viral concentration within the media is also significant in determining viral stability. Relatively high titres persist longer than less concentrated inoculums. This has been demonstrated with the enveloped influenza virus [39,40], and SARS-CoV-1 [41]. The latter virus persisted on paper for 3–6 h at 10^5^ TCID_50_/mL but for <5 min at 10^4^ TCID_50_/mL. Thus, a 10-fold increase in initial titre led to a >36-fold increase in persistence. Importantly, titres of ~10^3.5^ TCID_50_/mL closely resemble concentrations found in the average COVID-19 patient’s upper and lower respiratory tract [14,22], so data obtained from such inoculums may be more representative of the real-world behaviour of SARS-CoV-2. However, in extreme cases patients’ viral loads peak at >10^6^ TCID_50_/mL [42,43]. Therefore, studies that inoculated substrates with high-titre droplets effectively simulate the worst-case scenario. The effect of droplet size on SARS-CoV-2 stability warrants further investigation. Although Biryukov et al. inoculated substrates with droplets ranging from 1–50 μL and determined volume was insignificant, more research is required to validate this claim [34].

## 5. Effect of Temperature, Relative Humidity, and UV Irradiation on SARS-CoV-2 Persistence

The stability of enveloped viruses is usually enhanced at low temperatures and/or RH levels of <50% [2,44]. Although there are exceptions to this, coronavirus persistence rates follow the common trend [15,18,23,34,35,36,37].

The half-life of SARS-CoV-2 dried in a clinically relevant matrix on polystyrene was 3.3–5.8 h at 4 °C and 40% RH, 3.1 h at 21 °C and 40% RH, and 1.5 h at 27 °C and 85% RH [23]. In a separate study, the virus’s half-life dried on non-porous surfaces at RT was 15.33 h at 20% RH, 11.52 h at 40%, and 8.33 h at 80% RH [34]. Additional studies demonstrate the same relationship between SARS-CoV-2 persistence and temperature, although different RH levels were not assayed [15,18]. Anomalously, Kratzel et al. reported that SARS-CoV-2 was more stable on stainless steel at 30 °C than at 4 °C. However, the data were extremely variable [20]. Like SARS-CoV-2, the persistence rates of the closely related SARS-CoV-1 and MERS-CoV pathogens were inversely related to temperature and RH level [36,37].

The MERS-CoV pathogen persisted for 48–72 h (RT; 40% RH), 24–48 h (30 °C; 30% RH), and 8–24 h (30 °C; 80% RH). The stability of another enveloped virus, influenza A type H1N1, was assayed for comparison and this virus could not be detected after four hours regardless of ambient conditions, potentially indicative of coronaviruses’ unique stability compared to other enveloped viruses [37]. Chan et al. noted that SARS-CoV-1 remained viable for weeks under conditions akin to air-conditioned environments (RT; 40–50% RH) but was rapidly inactivated at 38 °C and 80–95% RH. This inactivation was even quicker when the RH level was increased to >95% [36]. The veterinary coronaviruses TGEV and MHV are like the SARS and MERS viruses, and it was reported that low temperatures and low RH levels (<50%) provide the most stability. However, the relationship between TGEV/MHV persistence and RH level was not monotonic. At RT, the animal coronaviruses appeared more stable at 80% RH versus 50% RH [35]. Importantly, the TGEV and MHV pathogens are gastrointestinal and hepatic disease-causing viruses, respectively, and may be adapted to different ambient conditions. 

While the studies noted directly above provide insight into the indoor conditions that promote or hinder coronavirus surface stability, outdoor settings present an additional factor: UV irradiation (following data not shown in Table 1). The effect of UVA (365 nm) and UVC (254 nm) irradiation on SARS-CoV-2 stock (initial titre = 5 × 10^6^ TCID_50_/mL) was explored by Heilingloh et al. after nine minutes of exposure to a UVA dose, a one-log titre reduction occurred. However, the virus was completely inactivated following a nine-minute exposure to a UVC dose [45]. Ratnesar-Shumate et al. inoculated stainless steel with SARS-CoV-2 (initial titre = ~ 10^3^ TCID_50_/mL) and assayed the effects of two UVB (280–315 nm) doses. A UVB dose corresponding to sunlight levels during the summer solstice at 40 °N latitude reduced the titre by 90% every 6.8 min. Sunlight levels corresponding to the winter solstice at 40 °N latitude inactivated 90% of the viral load every 14.3 min [46]. Comparably, no SARS-CoV-1 (initial titre = ~ 10^7^ TCID_50_/mL) was recovered after an hour of exposure to UVC (260 nm) irradiation [47]. These data align with the findings of Tseng and Li, who noted that single-stranded nucleic acid viruses (e.g., coronaviruses) were highly susceptible to UV inactivation [48]. Thus, indoor settings, especially those at low humidity levels, are likely SARS-CoV-2 transmission hotspots, but outdoor environments pose a much smaller risk. 

## 6. Effect of Material-Type on SARS-CoV-2 Persistence

A noteworthy oversight made by many researchers was the use of equivocal terminology like ‘plastic’ rather than specifying the type of plastic in their reports. Proper surface characterization was also lacking. Substrate porosity was often attributed to enhanced inactivation rates, but materials cannot simply be defined as porous or non-porous. Measured as a fraction, porosity describes the volume of empty space with respect to the total volume of a substrate [54]. Nonetheless, the present review will emulate such terminology for lack of better descriptions. 

With some exceptions, porous substrates appeared to inactivate SARS-CoV-2 and SARS-CoV-1 faster than non-porous substrates [14,15,16,17,19,22,25,26,41]. However, there was exceptional variation between viral stabilities on these materials, so the effects of these porous substrates are hard to generalize. Cotton- or cellulose-based materials usually attenuated the viruses quicker than any other substrate besides copper [14,15,16,17,19,22,25,26,41]. In fact, sometimes the drying process (t = < 1 h) on these materials was sufficient in fully inactivating relatively dilute inoculums [41]. The detrimental effects of cloth- and cellulose-based substrates on SARS-CoV stability align with the persistence of other enveloped viruses on similar materials [30,55,56]. On the contrary, SARS-CoV-2 demonstrated remarkable stability on surgical and N-95/N-100 masks despite being defined as porous. In three separate studies, it was shown that SARS-CoV-2 persistence was equal or greater on N-95 masks (>21 days) or surgical masks (>7 days) than on any other tested material [16,17,19]. 

The means through which porous materials seemingly inactivate coronaviruses may be multifaceted, but it is important to take note of experimental limitations. Porous materials can entrap viruses within their high-surface area matrix, which allows for many points of attachment [57]. Consequently, elution methods may be less effective on these substrates. Because most studies did not assay their elution efficiency, physical loss of virus may have been perceived as loss of infectivity. Therefore, it is possible that viruses are less likely to be transmitted through porous surfaces because they are trapped within the substrate, although not inactivated. The absorption/transport of media onto porous substrates may also be a factor, but coronaviruses are considerably stable in a dried state, so it is unclear how influential this factor is. 

The stability of SARS-CoV-2 was more consistent on non-porous surfaces. Apart from materials with intrinsic virucidal properties (e.g., copper), SARS-CoV-2 always persisted on the timescale of days at room temperature [14,15,16,17,18,19,20,21,22,23,24,25,26,34,49]. There may be a correlation between material wettability and SARS-CoV-2 stability. For example, the virus often persisted longer on polypropylene (hydrophobic) than on stainless steel, which is moderately hydrophilic [14,22]. Similarly, the virus was often more stable on plastic/stainless steel than on glass, which is considerably hydrophilic [19,21]. The surface of the SARS-CoV-2 virion is hydrophobic in nature [58]. Therefore, the hydrophobic effect may drive viral adsorption onto inert materials like polypropylene whereupon enhanced viral stability is achieved [59]. The adsorption of coronaviruses onto solid fractions in water matrices has been shown to enhance viral stability [60]. This may also partially explain the reduced persistence of SARS-CoV-2 on cotton- and cellulose-based materials, which are hydrophilic and thus repel viral adsorption. Notably, the correlation between non-porous surface wettability and SARS-CoV-2 stability did not always present itself and warrants further investigation [16,17,19,24,34,49].

Surprisingly, only one study was found that assayed the stability of SARS-CoV-2 on skin. The virus persisted for 96–168 h on swine skin, with a half-life of 3.5 h at RT and 40–50% RH. At a low temperature (4 °C), the virus was exceptionally stable, with a half-life of 46.8 h. Even at 37 °C, the virus remained viable for 8–24 h (half-life = 0.6 h) [15]. Swine and human skin are so alike, the former is commonly used for human allograft transplantations [61]. Thus, the work of Harbourt et al. speaks to the necessity of hand hygiene amidst the current pandemic [15].

## 7. Antiviral Surfaces and Future Material Innovation 

Amongst the many inoculated substrates, it is clear copper-containing surfaces demonstrated exceptional virucidal abilities to a variety of coronaviruses [14,22,33]. While SARS-CoV-2 persisted for days on most smooth surfaces, the virus persisted for 4–8 h on copper, with a half-life of 0.77 h [14,22]. Similarly, SARS-CoV-1 persisted for 72–96 h on polypropylene but only 8–24 h on copper [22]. On common household surfaces, HCoV-299E could be recovered for ≥5 days. However, the virus lost all infectivity after <30 min on alloys containing 90% copper. More dilute copper alloys were also effective, and their effectiveness was proportional to the percent-copper content [33]. Copper oxides may be even more effective towards the novel virus. Behzadinasab et al. created a widely applicable Cu_2_O/PU surface coating which could inactivate an extremely concentrated SARS-CoV-2 inoculum (~10^8^ TCID_50_/mL) in less than one hour. This remarkably effective coating was robust and retained its effectiveness after multiple inoculation/disinfection cycles and 13 days of soaking in water. Notably, in the same study it was found that monolayers of cationic polymer coatings (polyallylamine with primary amines or poly[diallyl dimethylammonium] chloride with quaternary ammonium) known to be effective against other viruses and bacteria were ineffective against SARS-CoV-2 [49].

The results of Pastorino et al. suggests that like copper, aluminum may also readily inactive SARS-CoV-2. Without the addition of BSA to the culture medium, the half-life of SARS-CoV-2 was only 2.5 h on aluminum versus >96 h on polypropylene [21]. The antiviral properties of aluminum are less documented than those of copper, but it has been shown that the B40-8 phage, adenovirus, and poliovirus remain viable on aluminum for a shorter time than on other impervious substrates [62,63]. Sizun et al. demonstrated HCoV-299E and HCoV-OC43 persisted for <12 h on aluminum, although similar inactivation rates were seen on latex gloves and cotton gauze [53]. Certainly, these findings suggest that more research should be conducted to discern the potential of aluminum surfaces to inactivate the novel virus. 

Surprisingly, no other studies were found which brought to light any other antiviral surface’s effectiveness towards SARS-CoV-2. This review presents considerable evidence that indirect transmission of SARS-CoV-2 is plausible, especially under indoor conditions away from sunlight, and the lack of research regarding anti-SARS-CoV-2 surfaces is concerning. Therefore, this review concludes by kindly prompting readers in the field to exploit the vast knowledge available on antimicrobial materials and experimentally extrapolate such to SARS-CoV-2. 

Antimicrobial surfaces can be divided into two categories: those that kill/inactivate pathogens (e.g., copper) and those that resist pathogen adhesion/retention. Regarding the first category, a wealth of knowledge is available on natural (e.g., herbs and extracts) and artificial (e.g., metal nanoparticles and graphene derivatives) substances with a broad range of virucidal properties towards many enveloped viruses [64,65,66]. As of right now (September 2020), every indoor public surface is serving as a likely site at which the virus can spread. Redesigning or modifying these surfaces such that the virus cannot remain infectious atop may be an answer to the unwavering spread of COVID-19. Alternatively, and regarding the second category of antimicrobial surfaces, anti-adhesion surfaces may prevent contamination from the get-go. If the virus could not attach to the material in the first place, the surface persistence of SARS-CoV-2 would not matter. Biomimetic surfaces like those modelled after nature’s lotus leaf have garnered attention in recent years due to their ability to resist contamination. The unique chemical and physical properties of these superhydrophobic surfaces allow for extreme water repellence, self-cleaning, and antifouling properties. Superhydrophobic surfaces with certain topographies could repel inoculating media and/or disallow SARS-CoV-2 adsorption [67]. 

The ideas listed above are few of many. A summary of antiviral materials/substances which may be effective towards SARS-CoV-2 is not within the scope of the present review, and this synopsis only aims to inspire readers to investigate abroad. Identifying and developing new anti-SARS-CoV-2 substrates/surface coatings would put researchers at the forefront of innovation to combat the spread of COVID-19. Moreover, as opposed to therapeutic drugs or vaccines, these innovative surfaces could provide preventative measures for future disease outbreaks to come. Material engineers, chemists, virologists, and every researcher in between may have worthy insight into ways of mitigating the current/potential future pandemic. The rampant spread of the novel virus can be battled with the rampant spread of knowledge and innovation. 

## 8. Concluding Remarks

The novel virus is durable and persists on many dry surfaces on the timescale of days. Consequently, indirect transmission of SARS-CoV-2 is likely. Notwithstanding decreases in population density, the virus will persist on high-touch surfaces long enough to spread to new individuals. This is especially true in cold, dry climates as SARS-COV-2 persistence is inversely related to temperature and humidity. Sunlight may rapidly inactivate the virus, indicating that indirect transmission may predominately occur indoors. Importantly, SARS-CoV-2 is considerably stable on human skin, and thus hand hygiene is of the utmost importance in mitigating SARS-CoV-2 indirect transmission. Identifying and developing new anti-SARS-CoV-2 substrates/surface coatings would put researchers at the forefront of innovation to combat the spread of COVID-19. Future research on SARS-CoV-2 persistence should be conducted with clinically relevant deposition media and titres. The representativeness of other coronaviruses’ stabilities on dry surfaces is unclear and warrants further investigation.

## Figures and Tables

**Table 1:** The stability of SARS-CoV-2 and other coronaviruses on dry surfaces. Articles are listed in reverse chronological order based on when they were published. Durations of infectivity are presented as ranges. The lower range value corresponds to the final sampling point at which infectious virus could be recovered from the surface. The upper range value corresponds to the first sampling point at which the quantity of infectious virus was below the lower limit of detection (LOD). The approximate titre reductions are in units TCID_50_/mL unless otherwise noted; the first value corresponds to the initial titre and the proceeding value corresponds to the LOD. Media is the matrix used to dry the viruses on the surfaces. Type is the name of the strain or isolate of the virus. Relative humidity: RH.

Ref.	Virus	Substrate	Time	Half- Life (Hours)	Titre (log)	Temp. (°C)	RH (%)	Media	Vol. (µL)	Type
[23]	SARS-CoV-2	Polystyrene	~36 h	3.3	5–0.5	4	40	Nasal Mucus	50	USA-WA1/2020
			~24 h	3.1		21				
			~12 h	1.5		27	85			
			~48 h	5.8		4	40	Sputum		
			~24 h	3.1		21				
			~12 h	1.5		27	85			
[18]	SARS-CoV-2	Stainless Steel	72–96 h	n/a	6.2–0.8	7	65	Culture Media	50	BetaCoV/France/IDF0571/2020)
			48–72 h			25				
						7		Artificial Saliva/ Mucus		
			30–48 h			25				
	HCoV-229E		>96 h		7.1–0.8	7		Culture Media		ATCC-VR-740
						25				
						7		Artificial Saliva/ Mucus		
			30–48 h			25				
	FCoV		>96 h		6.8–0.6	7		Culture Media		RVB-1259
			72–96 h			25				
			>96 h			7		Artificial Saliva/Mucus		
			24–30 h			25				
[26]	SARS-CoV-2	USB Cassette Talking Book (Acrylonitrile Butadiene Styrene, Specific Blend), Rigid Plastic Storage Container (high-density Polyethylene), Plexiglas (Acrylic)	>5 d	n/a	4.7–1.1	22	30–50	Culture Medium	100	USA-WA1/2020
		DVD (Polycarbonate), Flexible Plastic Storage Bag (Low-Density Polyethylene)	4–5 d							
[25]	SARS-CoV-2	Braille Paper Pages, Glossy Book Pages, Children’s Board Book (all Stacked)	3–4 d	n/a	5.26–1.1	22	30–50	Culture Medium	100	USA-WA1/2020
		Magazine Pages (Stacked)	>4 d							
		Archival Folders (Stacked) (Materials were Stacked after Inoculation to Mimic Storage Conditions in Libraries)	1–2 d							
[49]	SARS-CoV-2	Cu_2_O/Polyurethane (PU) Coating on Glass	0–1 h	n/a	7.8–2	22–23	60–70	Culture Medium	5	n/a
		Cu_2_O/PU on Stainless Steel	1–3 h							
		PU Coating on Glass, Polymeric Cation Coating on Glass, Bare Stainless Steel, Bare Glass	>24 h							
[15]	SARS-CoV-2	Swine Skin	>336 h	46.8	* 4.5–0.1	4	40–50	Culture Medium	50	USA-WA1/2020
			96–168 h	3.5		22				
			8–24 h	0.6		37				
		USD 1 Bank Note (25% Linen and 75% Cotton)	168–336 h	33.2		4				
			8–24 h	1.3		22				
			4–8 h	0.4		37				
		USD 20 Bank Note (25% Linen and 75% Cotton)	168–336 h	15.9		4				
			24–72 h	1.1		22				
			8–24 h	0.6		37				
		Clothing (35% Cotton and 65% Polyester)	96–168 h	33.7		4				
			4–8 h	1.0		22				
				0.2		37				
[24]	SARS-CoV-2	Hardback Book Cover, Paperback Book Cover, DVD Case	1 h–1 d	n/a	5.5–1.1	22	30–50	Culture Medium	100	USA-WA1/2020
		Plain Paper, Plastic Protective Cover	1–3 d							
[20]	SARS-CoV-2	Metal	192–214 h	12. 9	7.3–2	4	30–40	Culture Medium w/0.3% BSA	n/a	Munchen-1.1/2020/929
			120–144 h	9.1		RT				
			>214 h	17.9		30				
[34]	SARS-CoV-2	Stainless Steel, Acrylonitrile Butadiene Styrene Plastic, Nitrile Rubber Gloves	>48 h	15.33	2–0.2	24	20	Simulated Saliva	1–50	USA-WA1/2020
			24–48 h	11.52			40			
				9.15			60			
			n/a	8.33			80			
				6.11		28	40			
			24–48 h	7.33		35	20			
			9–24 h	7.52			40			
			3–9 h	2.26			60			
[16]	SARS-CoV-2	Nitrile Medical Examination Gloves	7–14 d	n/a	7.88–0.5	20	35–40	Tripartite Soil Load w/Mucin, BSA, and Tryptone	10	hCoV-19/Canada/ON-VIDO-01
		Reinforced Chemical Resistant Gloves	4–7 d							
		N-95 Mask	>21 d							
		N-100 Mask, Tyvek ® Coveralls, Plastic from Face Shields, Stainless Steel	14–21 d							
		Heavy Cotton	4 h–1 d							
[17]	SARS-CoV-2	Plastic	>7 d	0.57, 16.38	6–1.5	25–27	35	Culture Medium	50	BetaCoV/Beijing/AMMS01/2020
		Stainless Steel		0.83, 22.88						
		Glass		0.84, 22.30						
		Surgical Mask		0.64, 19.07						
		Ceramics		0.51, 21.71						
		Latex Gloves		0.54, 10.28						
		wood		0.20, 21.41						
		Cotton clothes	4–5 d	0.17, 22.72						
		Paper	5–7 d	4.75						
[14]	SARS-CoV-2	Polypropylene	~50 h	n/a	3.5–n/a	21–23	40	Culture medium	50	n/a
		Stainless steel	~30							
		Cardboard	~24 h							
		Copper	~5 h							
[21]	SARS-CoV-2	Polypropylene	>96 h	>96	6–0.5	19–21	45–55	Culture Medium w/1.8 g/L FBS	50	n/a
								FBS Culture Medium + 10 g/L BSA		
		Glass	24–48 h	17				Culture Medium w/1.8 g/L FBS		
			>96 h	>96				FBS Culture Medium + 10 g/L BSA		
		Aluminum	2–4 h	2.5				Culture Medium w/1.8 g/L FBS		
			>96 h	>96				FBS Culture Medium + 10 g/L BSA		
[19]	SARS-CoV-2	Outer Surgical Mask	>7 d	1.4, 23.9	7.8–2	22	65	Culture Medium	5	n/a
		Inner Surgical Mask	4–7 d	1.0, 9.9						
		Plastic		1.6, 11.4						
		Stainless Steel		0.3, 14.7						
		Glass	2–4 d	1.2, 4.8						
		Banknote		0.9, 7.9						
		Wood, Cloth	1–2 d	n/a						
		Printing/ Tissue Paper	1–3 h							
[22]	SARS-CoV-2	Plastic	72–96 h	6.81	3.5–0.6	21–-23	65	Culture Medium	50	nCoV-WA1-2020
		Stainless Steel	48–72 h	5.63						
		Cardboard	24–48 h	3.46						
		Copper	4–8 h	0.77	3.5–1.5					
	SARS-CoV-1	Plastic	72–96 h	7.55	3.5–0.6					Tor 2
		Stainless Steel	48–72 h	4.16						
		Cardboard	8–24 h	0.59						
		Copper		1.5	3.5–1.5					
[50]	HCoV-299E	Plastic, Glass, Stainless Steel	>7 d	n/a	*6–3.5	24	50	Culture Medium	20	VR-740
[33]	HCoV-299E	Stainless Steel, Teflon (Polytetrafluoroethylene), Polyvinyl Chloride, Ceramic, Glass, Silicone Rubber	≥5 d	n/a	* 4.5–1.5	21	30–40	Lung Cell Lysate	20	n/a
		Brass (90% Copper)	<30 min							
		Brass (70% Copper)	≤45 min							
		Copper Nickel (90% Copper)	<30 min							
		Copper Nickel (70% Copper)	≤125 min							
[37]	MERS-CoV	Stainless Steel, Plastic	48–72 h	0.44–0.97	6–n/a	20	40	Culture Medium	100	HCoV-EMC/2012
			24–48 h			30	30			
			8–24 h			30	80			
[36]	SARS-CoV-1	Plastic	~28 d	n/a	7–2	22–25	40–50	Culture Medium	10	HKU39849
						28	80–> 95			
						33				
			~24 h		7–5	38	80–89			
					7–3.5	38	>95			
[35]	TGEV	Stainless Steel	>28 d	n/a	** 6.5–0.5	4	20	Culture Medium	10	n/a
							50			
							80			
						20	20			
			>3 d				50			
			>14 d				80			
			96–120 h			40	20			
			6–12 h				50			
			4–6 h				80			
	MHV		>28 d			4	20			
							50			
							80			
						20	20			
			4–5 d				50			
			10–11 d				80			
			>120 h			40	20			
			>24 h				50			
			4–6 h				80			
[51]	TGEV	N95 Respirator, Contact Isolation Gowns	>24 h	n/a	** >3–n/a	20	50	Culture Medium	10	n/a
		Latex Gloves, Nitrile Gloves, Hospital Scrub	4–24 h							
[52]	HCoV-NL63	Latex Gloves, Thermometer Caps, Stethoscopes, Plastic Tables	<1 h	n/a	n/a	n/a	n/a	Culture Medium	n/a	n/a
[31]	SARS-CoV-1	Polystyrene	6–9 d	n/a	6.5–1.5	21–25	n/a	Culture Medium	500	FMM-1
								Culture Medium + 10% FCS		
	HCoV-299E		48–72 h					Culture Medium		n/a
			24–48 h					Culture Medium + 10% FCS		
[41]	SARS-CoV-1	Polyethylene Coated Gown	>2 d	n/a	6–n/a	20	n/a	Culture Medium Diluted in PBS	5	GVU6109
			1–2 d		5–n/a					
			1–2 h		4–n/a					
		Paper	1–2 d		6–n/a					
			3–6 h		5–n/a					
			<5 m		4–n/a					
		Cotton Gown	1–2 d		6–n/a					
			1–2 h		5–n/a					
			5 min–1 h		4–n/a					
[47]	SARS-CoV-1	Metal, Cloth, Filter Paper	>120 h	n/a	6.5–n/a	20	n/a	Culture Medium	300	P9
		Plastic, Glass, Press Paper, Wood	96–120 h							
		Mosaic	72–96 h							
[53]	HCoV-299E	Aluminum, Cotton Gauze, Latex Gloves	6–12 h	n/a	5.5–n/a	21	55–70	Culture Medium	10	n/a
	HCoV-OC43		2–3 h							

Half-lives with two values are indicative of biphasic decay rates. Human coronavirus: HCoV; Feline coronavirus: FCoV; Middle Eastern respiratory coronavirus: MERS-CoV; Transmissible gastroenteritis virus: TGEV; Murine hepatitis virus: MHV; Bovine serum albumin: BSA; Fetal calf serum: FCS; Fetal bovine serum: FBS; * titre reductions are in units PFU/mL; ** titre reductions are in units MPN/mL.
