# Correction: Bueckert et al. Infectivity of SARS-CoV-2 and Other Coronaviruses on Dry Surfaces: Potential for Indirect Transmission. *Materials* 2020, *13*, 5211

**DOI:** 10.3390/ma14112816

**Published:** 2021-05-25

**Authors:** Max Bueckert, Rishi Gupta, Aditi Gupta, Mohit Garg, Asit Mazumder

**Affiliations:** 1 Department of Biochemistry & Microbiology, University of Victoria, 3800 Finnerty Road, Victoria, BC V8P 5C2, Canada; 2 Department of Civil Engineering, University of Victoria, 3800 Finnerty Road, Victoria, BC V8P 5C2, Canada; mgarg@uvic.ca; 3 Mearns Centre for Learning—McPherson Library, University of Victoria, 3800 Finnerty Road, Victoria, BC V8P 5C2, Canada; aditig@uvic.ca; 4 Department of Biology, University of Victoria, 3800 Finnerty Road, Victoria, BC V8P 5C2, Canada; mazumder@uvic.ca

The authors wish to make the following corrections to this paper [1]:

1. Throughout the paper’s text and in the table, “HCoV-299E” is referred to a few times. However, this has been changed and referred to as “HCoV-229E”.
2. Citation [60] (Ye, Y.; Ellenberg, R.M.; Graham, K.E.; Wigginton, K.R. Survivability, Partitioning, and Recovery of Enveloped Viruses in Untreated Municipal Wastewater. *Environ. Sci. Technol.* **2016**, *50*, 5077–5085, doi:10.1021/acs.est.6b00876.) should be changed to the following article “Gundy, P.M.; Gerba, C.P.; Pepper, I.L. Survival of Coronaviruses in Water and Wastewater. *Food Environ. Virol.* **2009**, *1*, 10”.

The authors would like to apologise for any inconvenience caused to the readers by these changes.
